# Phytochemical Screening on Phenolic, Flavonoid Contents, and Antioxidant Activities of Six Indigenous Plants Used in Traditional Thai Medicine

**DOI:** 10.3390/ijms241713425

**Published:** 2023-08-30

**Authors:** Tanawuth Tiranakwit, Wimonwan Puangpun, Kawintra Tamprasit, Natthapong Wichai, Sirithon Siriamornpun, Tarapong Srisongkram, Natthida Weerapreeyakul

**Affiliations:** 1 Program of Pharmaceutical Sciences, Graduate School, Faculty of Pharmaceutical Sciences, Khon Kaen University, Khon Kaen 40002, Thailand; tiranakwit_t@kkumail.com; 2 Program of Doctor of Pharmacy, Faculty of Pharmaceutical Sciences, Khon Kaen University, Khon Kaen 40002, Thailand; wimonwn_p@kkumail.com; 3 Human High Performance and Health Promotion Research Institute, Khon Kaen University, Khon Kaen 40002, Thailand; ta.kawintra@kkumail.com (K.T.); tarasri@kku.ac.th (T.S.); 4 Department of Pharmaceutical Sciences, Faculty of Pharmacy, Mahasarakham University, Maha Sarakham 44150, Thailand; nuttapong.w@msu.ac.th; 5 Research Unit of Thai Food Innovation, Department of Food Technology and Nutrition, Faculty of Technology, Mahasarakham University, Maha Sarakham 44150, Thailand; sirithon.s@msu.ac.th; 6 Division of Pharmaceutical Chemistry, Faculty of Pharmaceutical Sciences, Khon Kaen University, Khon Kaen 40002, Thailand

**Keywords:** antioxidant, phenolics, flavonoids, indigenous plants, heatmap, principal component analysis

## Abstract

The antioxidant activity of a traditional Thai formula has been studied and compared to each plant. The formula comprised the roots of *Caesalpinia digyna* Rottler, *Huberantha cerasoides* (Roxb.) Benth), *Oxyceros horridus* Lour, *Antidesma ghaesembilla* Gaerth, *Combretum quadrangulare* Kurz, and *Ziziphus cambodiana* Pierre. The stem was also studied in comparison. The ethanolic extract from each plant part and the mixed plants mimicking the traditional formula were prepared and investigated for antioxidant capability in vitro via DPPH radical scavenging and ferric-reducing antioxidant power assays. The phytochemical constituents were determined by chemical screening, total phenolic (TPC) and flavonoid contents (TFC), and high-performance liquid chromatography. The relationship between antioxidant activity and the contributed phytochemicals was determined using correlation analysis and principal component analysis (PCA). Results showed that extracts from both parts of the plant formula showed the highest antioxidant activity compared to a single plant extract. Among the six plants, *C. digyna* exhibited the highest TPC and antioxidant activity. TPC had a strong positive correlation with antioxidant activity. PCA revealed that gallic acid contributed to the antioxidant activity. In conclusion, the ethanolic extracts of the traditional formula and *C. digyna* have the potential for further chemical characterization and study related to antioxidant activity.

## 1. Introduction

Many traditional plants have led to the discovery of numerous leading compounds and pharmaceuticals [1]. Traditional medicines are widely used in Thailand as a remedy for various illnesses [2,3,4,5,6]. Traditional botanical medicines have been used by pregnant women in the local northeastern area of Thailand [7,8,9,10]. One traditional Thai formula has been used for puerperium care after parturition. This formula was based on an interview with a local northeastern Thai folklore medicine practitioner. It contains the root of six plants, including *Caesalpinia digyna* Rottler (CD), *Huberantha cerasoides* (Roxb.) Benth (HC), *Oxyceros horridus* Lour (OH), *Antidesma ghaesembilla* Gaerth (AG), *Combretum quadrangulare* Kurz (CQ), and *Ziziphus cambodiana* Pierre (ZC) (Table 1). The plant in the formulation used in this study contained CD:HC:OH:AG:CQ:ZC in a ratio of 1:1:0.5:1:1:1. This traditional Thai medicine has been taken orally after delivery to nourish the blood, to increase blood flow and secretion of amniotic fluid, promote galactagogue, and reduce fatigue.

Oxidative stress is the phenomenon of the imbalance between the generation and accumulation of reactive oxygen species (ROS) in cells and tissues (for example, the oxidation of lipids) and the ability of a biological system to get rid of these reactive products in cells and tissue. Oxidative stress has been involved in the pathophysiology of many diseases, reproductive and pregnancy disorders, labor, and birth [11,12,13]. Pregnancy has been known to increase oxidative stress by a normal systemic inflammatory response leading to a high circulating ROS and reactive nitrogen species (RNS). Both of them play a pivotal role as secondary messengers in many intracellular signaling cascades and possess important effects on pathological processes in pregnant women [14]. The increase in oxidative stress during the labor process was associated with an increase in the NF-κB pathway, which modulated pro-inflammatory cytokines [15]. Moreover, postpartum condition is implicated in oxidative stress and developing many continuous symptoms after pregnancy, such as postpartum diabetes, depression, and postpartum hemorrhage [16,17,18,19].

According to the uses of traditional Thai medicine, it was hypothesized that this formula may be used as an antioxidant to overcome oxidative stress after the postpartum period. However, there is no proof that it can be used as an antioxidant therapy. The antioxidant activity of the single plant extracts used in the formula of traditional Thai medicine has been reported previously [20,21,22,23,24,25,26,27], however, with various methods. This requires a thorough study to investigate the antioxidant activity of the formula, compared to each plant, and their phytoconstituent profiles. The same extraction method was exploited for the root and stem of each plant, and a formula containing six mixed plants. This formula followed the local wisdom and knowledge of traditional Thai medicine. The phytochemical constitution of each plant extract and the antioxidant activity were investigated. In addition, their relationship was analyzed by adapting the correlation and principal component analysis. The obtained information could support future pharmacological activity studies based on traditional Thai medicine and the standardization of the raw material in further studies or during natural product development.

## 2. Results

### 2.1. Extraction Yield

The extraction yield was calculated from the percentage of extract weight per weight of dry plant powder. This value reflects the extraction conditions. The respective extraction yield of the dry extract obtained from all plants is shown in Table 1. The extraction yield ranged between 1.56 and 15.82% *w*/*w*. The extraction yield from the root is generally higher than that from the stem in all plants. The extract of *C. digyna* root and stem showed the highest percentage of yield (15.82 and 6.17 % *w*/*w* dry plant, respectively). On the other hand, the *O. horridus* stem showed the lowest percentage of yield at 1.56% *w*/*w*.

### 2.2. Screening of Phytochemical Constituents

**Figure 1:**
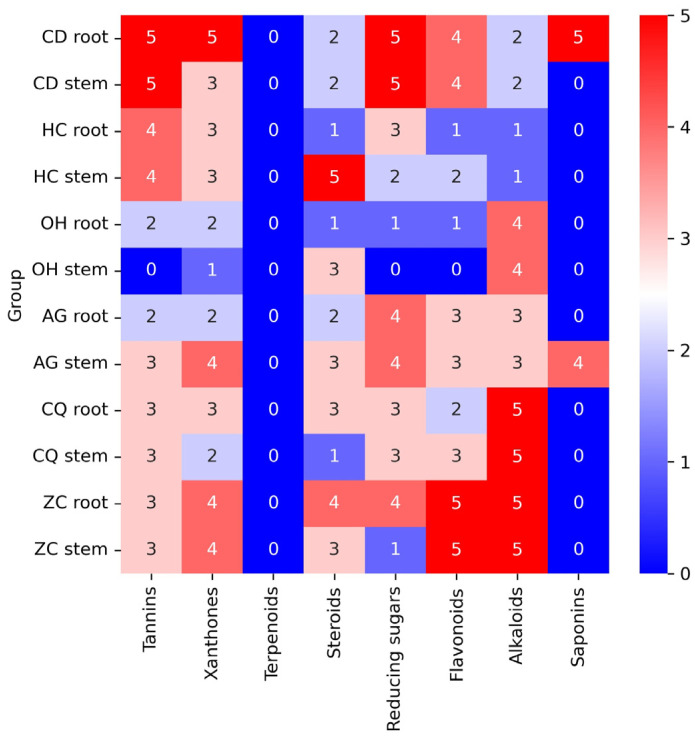
The heatmap shows the evaluation of phytochemical constituents in plant extracts. The blue to red color indicates the amount from the absence (0 score) to the highest presence (5 score) of the phytochemicals in each sample. The color scale is shown on the right side of the heatmap. CD: *Caesalpinia digyna*, HC: *Huberantha cerasoides*, OH: *Oxyceros horridus*, AG: *Antidesma ghaesembilla*, CQ: *Combretum quadrangulare*, ZC: *Ziziphus cambodiana*.

The phytochemical constituents were screened to evaluate the groups of compounds contained in each plant extract, which might be attributed to the bioactivity studied. The presence of phytochemicals constituted in the extracts was visually observed based on the color intensity, precipitation, and height of foam formation compared to the control (without the crude extract). The presence of tannin was indicated by a dark green or blue-black precipitate, xanthones as a yellow precipitate, terpenoids as a gray color, steroids with red color in the lower layer, reducing sugar as a brick-red precipitate, flavonoids as yellow color, alkaloids as a reddish-brown precipitate with turbidity, and saponins as a formation of permanent foam at 25 °C (Supplementary Table S1). The contents of tannins, xanthones, terpenoids, steroids, reducing sugar, flavonoids, alkaloids, and saponins in 6 Thai plants extracted from root and stem (12 samples) are shown in the heatmap (Figure 1). Each phytochemical content in the extracted sample was assigned a value between 0 and 5 (blue to red), where 0 indicates an absence of the compound and numbers 1 to 5 indicate the presence of the compound from the lowest to the highest amount observed in comparison to the control (without the crude extract).

The highest amount of tannins (5 score) was found in the root and stem of *C. digyna*, followed by the root and stem of *H. cerasoides* (4 score), the stem of *A. ghaesembilla,* and both parts of *C. quadrangulare*, and *Z. cambodiana* (3 score), the root of *O. horridus*, and *A. ghaesembilla* (2 score). The stem of *O. horridus* was not observed to contain tannins (0 score).

The highest xanthone contents were observed in the root of *C. digyna* (5 score), more than the stem of *A. ghaesembilla* and both parts of *Z. cambodiana* (4 score); the stem of *C. digyna*, both parts of *H. cerasoides*, the root of *C. quadrangulare* (3 score); and the root of *O. horridus* and *A. ghaesembilla*, and stem of *C. quadrangulare* (2 score). The lowest xanthone content (1 score) was in the stem of *O. horridus*.

For steroids, the highest amount (5 score) was in the stem of *H. cerasoides*, followed by the root of *Z. cambodiana* (4 score), the stem of *O. horridus*, *A. ghaesembilla*, and *Z. cambodiana*; the root of *C. quadrangulare* (3 score), the root of *A. ghaesembilla* and both parts of *C. digyna* (2 score), the root of *H. cerasoides* and *O. horridus*; and the stem of *C. quadrangulare* (1 score).

The highest reducing sugar was in both parts of *C. digyna* (5 score), followed by both parts of *A. ghaesembilla* and the root of *Z. cambodiana* (4 score), the root of *H. cerasoides*, and both parts of *C. quadrangulare* (3 score), the stem of *H. cerasoides* (2 score), and the root of *O. horridus* and the stem of *C. quadrangulare* (1 score). In contrast, *H. cerasoides* did not comprise any reducing sugar (0 score).

The highest flavonoid contents were found in both parts of *Z. cambodiana* (5 score), followed by both parts of *C. digyna* (4 score), both parts of *A. ghaesembilla* and the stem of *C. quadrangulare* (3 score), *H. cerasoides* stem and *C. quadrangulare* root (2 score), and *H. cerasoides* and *O. horridus* roots (1 scor), while *O. horridus* stem did not contain any flavonoids (0 scores).

The highest alkaloid content was observed in both parts of *C. quadrangulare* and *Z. Cambodiana* (5 score) more than both parts of *O*. *horridus* (4 score), both parts of *A*. *ghaesembilla* (3 score), both parts of *C. digyna* (2 score), and both parts of *H. cerasoides* (1 score).

Saponins were found in only two plants. The highest foam formation was in the root of *C. digyna* (5 score), followed by the stem of *A. ghaesembilla* (4 score). Remarkably, none of the plants studied contained terpenoids.

### 2.3. HPLC Analysis for Phenolic and Flavonoid Contents

HPLC analysis was used to determine phenolic and flavonoid contents in the plant extract. Three groups of ten standard compounds were quantified and used as a reference marker. They were (i) hydroxybenzoic acids, including gallic acid (1), protocatechuic acid (2), vanillic acid (3), and syringic acid (5), (ii) hydroxycinnamic acids, including caffeic acid (4), ferulic acid (6), and cinnamic acid (7), and (iii) flavonoids, including rutin (8), quercetin (9), and apigenin (10). The HPLC chromatograms of the standard compounds, each plant extract, and the formula extract are shown in Supplementary Figures S1–S8, respectively. For compound identification, the retention times of the standard compounds were matched with the retention times in the plant extracts at each detected wavelength (Supplementary Table S2). The phytochemical contents in each plant and plant formula were calculated from the peak height of the extract compared to the peak height of standard compounds.

Ten reference phenolic and flavonoid compounds were selected because they are commonly presented in various plants and have been widely investigated for qualitative and quantitative analysis using HPLC [28,29,30]. Nevertheless, only seven compounds (gallic acid (1), protocatechuic acid (2), vanillic acid (3), cinnamic acid (7), rutin (8), quercetin (9), and apigenin (10)) were found in the plant in our study. The absence of additional compounds may be related to the natural variety of these chemicals in plants.

Results show that gallic acid was found in an extract from the six mixed plant extracts and in the extracts of *C. digyna*, *A. ghaesembilla*, and *C. quadrangulare* (Table 2). A significantly higher gallic acid content was found in a mixed stem extract, *C. digyna*, and *A. ghaesembilla* than in the root. The stem extract from *C. digyna* had a higher gallic acid content (1368.1 ± 4.0 µg/g extract) than a mixed stem extract (617 ± 6.0 µg/g extract), *A. ghaesembilla* (321.8 ± 0.52 µg/g extract), and *C*. *quadrangulare* (151.9 ± 2.3 µg/g extract), respectively. The root extract of *C. quadrangulare* has a significantly higher gallic acid content than in its stem. Comparing the gallic acid in the root extract, *C. digyna* contained a higher gallic acid content (735.4 ± 1.3 µg/g extract), followed by a mixed root extract (401.7 ± 4.3 µg/g extract), *C. quadrangulare* (376.2 ± 3.9 µg/g extract), and *A. ghaesembilla* (179.1 ± 1.9 µg/g extract), respectively.

Protocatechuic acid was only found in the root of *C. quadrangulare* (131.4 ± 4.5 µg/g extract). Vanillic acid was detected in the stem part of both *C. digyna* and a mixed stem extract, in the amounts of 142.8 ± 0.5 µg/g extract and 98.3 ± 1.8 µg/g extract, respectively. Cinnamic acid was in the stem of *H. cerasoides* (691.3 ± 4.7 µg/g extract), a mixed stem ex-tract (207.2 ± 4.1 µg/g extract), and the root of *O. horridus* (109.6 ± 1.6 µg/g extract).

The extracts from the root and stem of *C. quadrangulare* and a mixed plant extract were composed of rutin, while only the root extract of *H. cerasoides* was composed of rutin. The extracts from the same root part of *C. quadrangulare* had a higher rutin content (1083.4 ± 17.9 µg/g extract) than *H. cerasoides* (111.1 ± 2.9 µg/g extract), and a mixed root extract (95.5 ± 2.6 µg/g extract). The stem extract of *C. quadrangulare* had higher rutin (1373.1 ± 75.6 µg/g extract) than a mixed stem extract (139.0 ± 3.6 µg/g extract).

Quercetin was detected in the stem extract of *H. cerasoides*, a mixed plant extract from the stem, and *C. digyna*. The extract from *H. cerasoides* had the highest quercetin (456.0 ± 17.3 µg/g extract), followed by a mixed stem extract (96.4 ± 0.8 µg/g extract), and *C. digyna* (87.01 ± 0.9 µg/g extract). Moreover, apigenin was detected in the stem of *H. cerasoides* (1344.3 ± 3.0 µg/g extract), a mixed stem extract (151.1 ± 0.8 µg/g extract), and the root of *C. digyna* (97.5 ± 2.9 µg/g extract).

### 2.4. Determination of Total Phenolic Content by Folin–Ciocalteu’s Reagent Method

The total phenolic contents (TPCs) in the ethanolic extract of six mixed plants and each plant are shown in Table 3. The extracts from six mixed plants, *C. digyna*, *A. ghaesembilla*, and *O. horridus*, contained a significantly higher TPC in the root than the stem. In contrast, *Z. cambodiana* and *C. quadrangulare* had a significantly higher TPC in the stem than the root. TPC in the root of *H. cerasoides* was not significantly different from its stem (138.4 ± 0.9 and 120.2 ± 2.1 mg of gallic acid equivalence (GAE)/g extract).

For comparing TPC per gram of root extract, a mixed root extract contained a higher TPC (1682.7 ± 19.8 mg of GAE) than the root of *C. digyna* (1085.8 ± 20.5 mg of GAE), *A. ghaesembilla* (396.41 ± 6.4 mg of GAE), *Z. cambodiana* (259.73 ± 8.2 mg of GAE), *C. quadrangulare* (190.5 ± 4.5 mg of GAE), *H. cerasoides* (138.4 ± 0.9 mg of GAE), and *O. horridus* (95.1 ± 2.9 mg of GAE), respectively.

Likewise, when comparing TPC per gram of stem extract, a mixed stem extract showed a higher TPC (1006.3 ± 8.6 mg of GAE) than *C. digyna* (661.4 ± 33.0 mg of GAE), *A. ghaesembilla* (328.8 ± 16.0 mg of GAE), *Z. cambodiana* (313.8 ± 7.4 mg of GAE), *C. quadrangulare* (222.3 ± 6.9 mg of GAE), and *H. cerasoides* (55.5 ± 1.0 mg of GAE).

### 2.5. Determination of Total Flavonoid Content

The total flavonoid contents (TFCs) of all single plant extracts and the extract of six mixed plants are shown in Table 3. Results show that a significantly higher TFC in the root extracts than their stem was detected in *A. ghaesembilla*, *C. digyna*, *C. quadrangulare*, and *O. horridus*. In contrast, a significantly higher TFC in the stem extract compared to the root was found in *Z. cambodiana* and *H. cerasoides*. In addition, TFC per gram extract from the root and stem of a mixed plant extract was not significantly different (23.0 ± 0.1 and 23.0 ± 0.4 mg of rutin). Comparing TFC per gram of the root extract, *Z. cambodiana* had the highest TFC (39.3 ± 0.8 mg of rutin), followed by *A. ghaesembilla* (30.0 ± 0.1 mg of rutin), *C. digyna* (24.7 ± 0.2 mg of rutin), a mixed plant extract (23.0 ± 0.1 mg of rutin), *C. quadrangulare* (15.4 ± 0.2 mg of rutin), *H. cerasoides* (11.5 ± 0.1 mg of rutin), and *O. horridus* (9.7 ± 0.1 mg of rutin), respectively.

When comparing TFC per gram of the stem extract, *Z. cambodiana* had the highest TFC (54.9 ± 1.0 mg of rutin), followed by *A. ghaesembilla* (27.1 ± 0.7 mg of rutin), a mixed plant extract (23.0 ± 0.4 mg of rutin), *C. digyna* (22.8 ± 0.3 mg of rutin), *H. cerasoides* (12.8 ± 0.1 mg of rutin), *C. quadrangulare* (10.4 ± 0.3 mg of rutin), and *O. horridus* (4.7 ± 0.2 mg of rutin). In addition, TFC per gram of a mixed stem extract (23.0 ± 0.4 mg of rutin) and *C. digyna* (22.8 ± 0.3 mg of rutin) was not significantly different (*p* > 0.05).

### 2.6. Determination of DPPH Radical Scavenging Effect

Antioxidant or radical scavenging agents can decolorize DPPH radicals (DPPH^•^). The antioxidant compound will interact with the DPPH radical (an oxidized form) to form DPPH-H (a reduced form) [31,32]. Once the DPPH radical is scavenged by the antioxidant, its color intensity will be decreased. Results are expressed as a half-maximal inhibitory concentration value (IC_50_) (µg/mL). The highest concentration of plant extract used in this assay was 500 µg/mL in order to obtain an IC_50_ value. Inactive means when a half-maximal inhibition was not obtained even when using a maximum concentration. The lower the IC_50_ value, the better the scavenging activity (Table 3). Gallic acid was a positive control, which inhibited the DPPH radical with an IC_50_ value of 3.4 ± 0.1 µg/mL.

When compared between parts, the extracts from a mixed plant extract and *C. digyna* possessed non-significantly different DPPH scavenging activity. Interestingly, the ranking from high to low DPPH radical scavenging activity was the same when considered in each part that *C. digyna* showed higher inhibition of DPPH radicals than *A. ghaesembilla*, *Z. cambodiana*, *C. quadrangulare*, *H. cerasoides*, and *O. horridus*, respectively. Among root extracts, a mixed root extract showed the highest DPPH scavenging activity. But among stem extracts, its activity was the second rank after the *C. digyna* stem extract.

When comparing the root extract, a mixed root extract and the *C. digyna* root extract showed the lowest IC_50_ values at 3.8 ± 0.2 and 7.8 ± 0.3 µg/mL, followed by *A. ghaesembilla* (32.3 ± 2.4 µg/mL) and *Z. cambodiana* (38.9 ± 0.7 µg/mL); *C. quadrangulare* (74.8 ± 1.1 µg/mL); *H. cerasoides* (172.9 ± 4.4 µg/mL); and *O. horridus* (257.1 ± 8.2 µg/mL).

Among the stem extract, *C. digyna* had the lowest IC_50_ value (5.8 ± 0.1 µg/mL), followed by a mixed stem extract (10.0 ± 0.3 µg/mL), *A. ghaesembilla* (45.6 ± 0.6 µg/mL), *Z. cambodiana* (47.8 ± 1.4 µg/mL), *C. quadrangulare* (57.8 ± 2.0 µg/mL), and *H. cerasoides* (267.3 ± 5.7 µg/mL). Non-significant differences in the activity were observed in a mixed stem extract and *C. digyna* and between *A. ghaesembilla* and *Z. cambodiana* (*p* > 0.05). The stem extract of *H. cerasoides* was inactive as it exerted no inhibitory activity, even using a con-centration as high as 500 µg/mL. The IC_50_ value, therefore, could not be calculated.

### 2.7. Determination of Ferric-Reducing Antioxidant Power (FRAP)

This assay of antioxidant activity was based on a compound’s reducing power. A potential antioxidant will reduce the ferric ion (Fe^3+^) to the ferrous ion (Fe^2+^). Ferric-reducing antioxidant power (FRAP) was reported as a millimolar FeSO_4_ equivalent per gram of dry extract (Table 3). The higher the FRAP value, the greater the reducing antioxidant power is. Results demonstrated that a mixed plant extract from root or stem possessed the highest FRAP values. Among single plant extracts, the ranking from high to low FRAP value was similar in each part that *C. digyna* displayed the highest reducing power compared to *A. ghaesembilla*, *Z*. *cambodiana*, and *C*. *quadrangulare*. The root extract of *O*. *horridus* showed higher reducing power than the root extract of *H. cerasoides*, and vice versa for the stem. The stem extract of *H*. *cerasoides* showed higher reducing power than the stem extract of *O*. *horridus*.

A mixed root extract and root extract from *C. digyna* had a significantly higher FRAP than their root stem. A non-significantly different reducing power between root and stem (per gram extract) was found in *A. ghaesembilla* (6.8 ± 0.4 and 7.0 ± 0.2 mM FeSO_4_ equivalent), *Z. cambodiana* (5.0 ± 0.1 and 5.6 ± 0.1 mM FeSO_4_ equivalent), *C. quadrangulare* (3.1 ± 0.2 and 3.0 ± 0.1 mM FeSO_4_ equivalent), *O. horridus* (0.3 ± 0.0 and 0.2 ± 0.0 mM FeSO_4_ equivalent), and *H. cerasoides* (1.8 ± 0.1 and 2.4 ± 0.0 mM FeSO_4_ equivalent).

Comparing the root extract, a mixed root extract had a significantly higher reducing power (32.3 ± 1.3 mM FeSO_4_ equivalent/g extract) than *C. digyna* (10.7 ± 0.5 mM FeSO_4_ equivalent/g extract), *A. ghaesembilla* (6.8 ± 0.4 mM FeSO_4_ equivalent/g extract), *Z. cambodiana* (5.0 ± 0.1 mM FeSO_4_ equivalent/g extract), and *C*. *quadrangulare* (3.1 ± 0.2 mM FeSO_4_ equivalent/g extract), *O. horridus* (2.2 ± 0.1 mM FeSO_4_ equivalent/g extract), and *H. cerasoides* (2.2 ± 0.1 mM FeSO_4_ equivalent/g extract). In addition, the root extracts of the pair between *C. quadrangulare* and *O. horridus* and between *O. horridus* and *H. cerasoides* were non-significantly different in reducing power (*p* > 0.05).

Comparing the stem extract, a mixed stem extract had a significantly higher reducing power (19 ± 1.0 mM FeSO_4_ equivalent/g extract), followed by *C. digyna* (9.3 ± 0.5 mM FeSO_4_ equivalent/g extract), *A*. *ghaesembilla* (7.0 ± 0.2 mM FeSO_4_ equivalent/g extract), *Z. cambodiana* (5.6 ± 0.1 mM FeSO_4_ equivalent/g extract), *C. quadrangulare* (3.0 ± 0.1 mM FeSO_4_ equivalent/g extract), and *H. cerasoides* (2.4 ± 0.0 mM FeSO_4_ equivalent/g extract), and *O. horridus* (1.3 ± 0.1 mM FeSO_4_ equivalent/g extract). The stem extract of the pair between *C. quadrangulare* and *H. cerasoides* and between *O. horridus* and *H. cerasoides* did not show significantly different reducing power (*p* > 0.05).

### 2.8. Correlation Analysis

**Figure 2:**
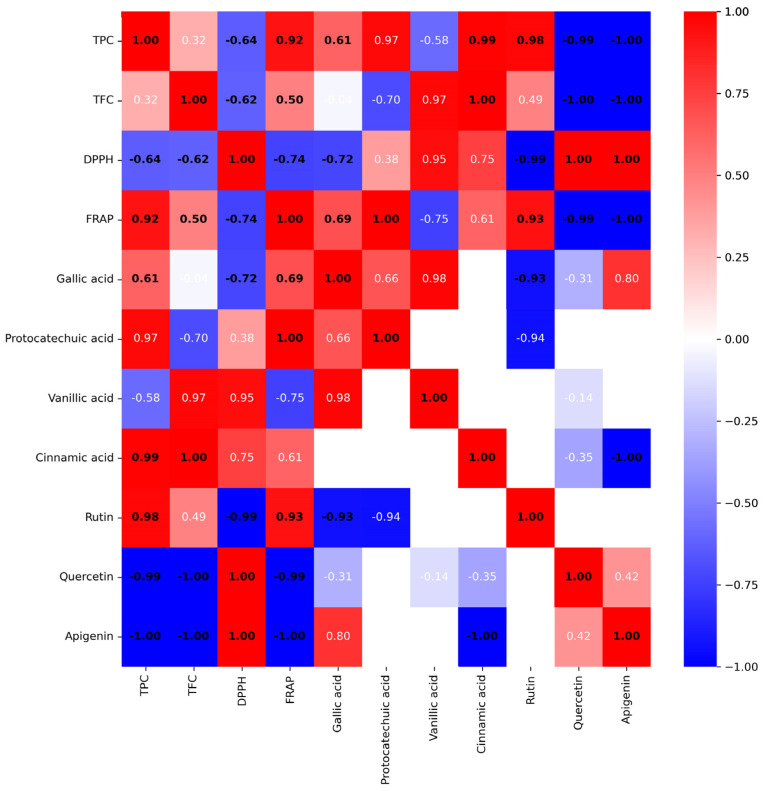
Pearson’s correlation analysis of phytochemical constituents and antioxidant activity of single plant extracts. The color bar of blue color to red color represents the correlation coefficient from a strong negative correlation (−1.00) to a very strong positive correlation (1.00). The color scale is shown on the right side of the heatmap. A significant correlation (*p* < 0.05) is labeled as a bold black number, and a non-significant correlation (*p* > 0.05) is labeled as a white number.

The correlation analysis between antioxidant activity and phytochemical constituents in single plant extracts is shown in Figure 2. The Pearson correlation coefficients (r) between antioxidant activity and phytochemical constituents were calculated and presented as numbers between −1 and 1 (blue to red), where zero indicated no correlation. The numbers were assigned for a different degree of correlation following a previous report [33] into a strong (0.80–1.00), a high (0.60–0.79), a moderate (0.40–0.59), a fair (0.20–0.39), and a weak (0.00–0.19) correlation. In addition, the significant correlation (*p* < 0.05) was also indicated as a bold black number, and a non-significant correlation (*p* > 0.05) is labeled as a white number (Figure 2).

The results obtained from TPC, TFC, DPPH, and FRAP assays were significantly well-correlated (Figure 2). TPC had a strong positive correlation with FRAP (r = 0.92) and had a high negative correlation with an IC_50_ value of DPPH assay (r = 0.64). TFC had a moderate positive correlation with FRAP value (r = 0.50) and a high negative correlation with IC_50_ value of the DPPH assay (r = 0.62). Results suggested that TPC and TFC contributed to an increase in antioxidant power due to a rise in FRAP value and a decrease in IC_50_ value (high activity) in the DPPH assay. Based on the high to strong correlation, TPC appears to have a more significant influence on antioxidant activity than TFC.

The Pearson correlation between the antioxidant activity and the quantitative results of polyphenols and flavonoids in the plant extracts by HPLC was also determined. Gallic acid shows a moderate negative correlation with the IC_50_ value of DPPH assay (r = −0.72) and a high positive correlation with FRAP value (r = 0.69). Rutin shows a very high negative correlation with the IC_50_ value of DPPH assay (r = −0.99) and a very high positive correlation with FRAP value (r = 0.93). The results indicated that gallic acid and rutin highly contributed to both mechanisms of antioxidant activity of the plant extract. Protocatechuic acid only significantly correlated to FRAP value with a high positive correlation (r = 1). In contrast, quercetin and apigenin possessed a strong negative influence on antioxidant activity.

### 2.9. Principal Component Analysis

The principal component analysis was conducted between the FRAP value and the IC_50_ value from the DPPH assay of 14 samples, including the stem and root extracts from six individual plants and the stem and root extracts from six mixed plants.

**Figure 3:**
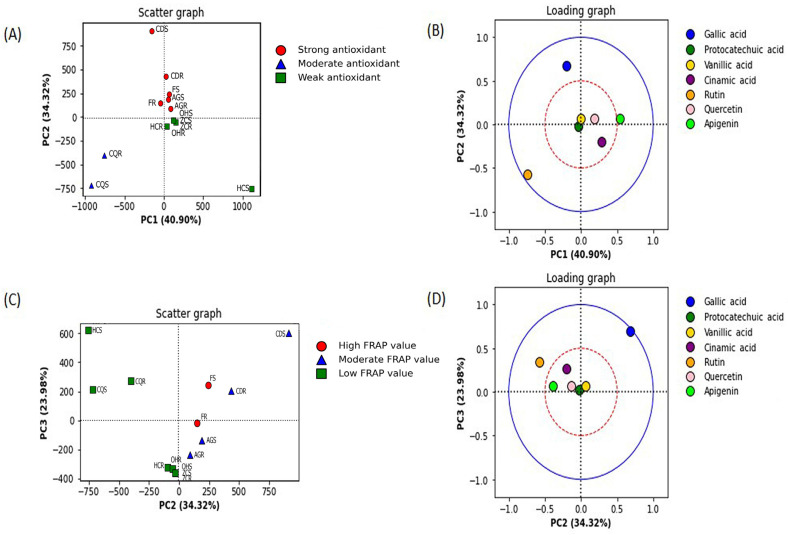
The principal component analysis of the antioxidant activity of the plant extracts. (**A**) Score plot between principal component 1 (PC1) and component 2 (PC2) to predict DPPH radical scavenging activity. The color of each dot indicated the groups of IC_50_ values. (**B**) Loading plot of principal component analysis between PC1 to PC2. (**C**) Score plot between principal component 2 (PC2) and component 3 (PC3) to predict FRAP value. The color of each dot indicated the level of FRAP value. (**D**) Loading plot of a principal component analysis between PC2 to PC3. The blue solid circle indicates the correlation between the components at 1.0, and the red dashed circle indicates the correlation between the components at 0.5.

The principal component analysis against DPPH radical scavenging activity (determined from the low IC_50_ value) was best described by PC1 and PC2 (Figure 3A). This antioxidant mechanism was classified into a strong (IC_50_ value ≤ 50 µg/mL), a moderate (IC_50_ value 51–100 µg/mL), and a weak (IC_50_ value >101 µg/mL) antioxidant. The phytochemical constituents that contributed to the radical scavenging activity were determined by the loading plot of PC1 and PC2 (Figure 3B). PC1 separates the extract groups with moderate scavenging activity from the groups with strong and weak activity (40.90%). PC2 separates the group of the extract with strong DPPH radical scavenging activity from the other groups (34.33%). In Figure 3, the blue solid (outer) circle indicates a strong correlation between the components ranging from 0.5 to 1.0, and the red dashed (inner) circle indicates a lower correlation between the components ranging from 0 to 0.5. Gallic acid and rutin are located outside the red circle (Figure 3B). The results indicated that gallic acid was the most contributed compound in the extract with strong radical scavenging activity, as it was well-correlated and appeared in the upper left quadrant. Rutin was well-correlated to the extract group with a moderate radical scavenging group (bottom left quadrant) (Figure 3B).

The principal component (PC) analysis against FRAP values revealed that the PC2 and PC3 best described the clustering of FRAP values (Figure 3C). The reducing power antioxidant activity was classified into three groups: a high FRAP value (≥11 mM FeSO_4_ equivalent/g extract), a moderate FRAP value (6–10 mM FeSO_4_ equivalent/g extract), and a low FRAP value (<6 mM FeSO_4_ equivalent/g extract). The phenolic compounds in the extracts were identified and quantified by the HPLC analysis and deter-mined for their contribution to the antioxidant activity based on the loading plot between PC2 and PC3 (Figure 3D). PC2 separates a group of the extract with a low FRAP value from the extract groups with a high to moderate value (34.32%). Gallic acid and rutin were located outside the red circle (Figure 3D), presenting a high loading correlation (>0.5). Gallic acid was the most influential compound in the extract with a high reducing power, as it appeared in the upper right quadrant of the loading plot. Rutin was the other influenced compound in the extract group with a low FRAP value, as shown in the upper left quadrant (Figure 3D).

The other detected compounds (i.e., protocatechuic acid, vanillic acid, cinnamic acid, quercetin, and apigenin) did not show a correlation to either mechanism of antioxidant activity, as they were not highly loaded (<0.5) (Figure 3B,D).

## 3. Discussion

Reactive oxygen species (ROS) are oxygen-containing radicals with one or more unpaired electrons or reactive oxygen-containing compounds with no unpaired electrons, such as hydrogen peroxide (H_2_O_2_) and singlet oxygen (^1^O_2_) [34]. ROS, such as hydroxyl radicals or superoxide anions, might cause cells to undergo oxidative stress and lead to damage to deoxyribonucleic acids (DNA), lipids [35], and proteins [36]. An excess of ROS can occur due to a high production of ROS from cell metabolisms or low levels of antioxidant compounds or enzymes [11]. The balancing of ROS is involved in many health systems, including the reproductive system. Pregnancy is the process that causes an increase in oxidative marker levels, and the oxidative marker slowly decreases back to an average level after giving birth [37].

Recently, natural antioxidants have been focused on due to the biodiversity of natural sources that allow the discovery of new active compounds [38]. The long history of traditional medicine’s uses also confirms their safety and biological activity, although this is still unproven. In the long term, the obtained information from the research study can support the use of medicinal plants, increase the value added by the local plants, and later improve the economic impact on the local people [39,40]. That is the reason why the study on antioxidants from medicinal plants has been of interest. In this study, the roots of six plants used in traditional Thai medicine were studied based on their historical use for women after giving birth, and it was found that they may be related to the amelioration of oxidative stress. The stem part was also collected and studied for comparison because it is more accessible and easier to collect than the root. In this study, ethanol was used for the extraction to mimic the traditional uses. In addition, ethanol is safely edible and leaves a safe-to-use extract. Ethanol has been used as a co-solvent for preparing herbal remedies, as mentioned in the monographs of traditional herbal medicine, including European [41], Indian [42], and Thai herbal Pharmacopeia [43].

Examples of numerous techniques include the oxygen radical absorbance capacity (ORAC) assay, the ferric-reducing/antioxidant power (FRAP) assay, the superoxide anion radical scavenging assay, DPPH radical scavenging capacity assay, and the metal chelating activity assay. These antioxidant activity modes require distinct techniques for measuring antioxidant capacity [44]. The FRAP assay has been used to evaluate the ability of an antioxidant to donate an electron [45,46]. The DPPH assay investigates the antioxidants’ ability to undergo electron transfer and hydrogen atom transfer reactions [47]. Many studies have reported a direct correlation between these two mechanisms of antioxidant activities of certain plant extracts and phenolic contents [45,46,48,49]. Various mechanisms of antioxidant activity of the phenolic group have been reported, including hydrogen atom transfer, single-electron transfer, sequential proton loss electron transfer, and transition metal chelation [50]. The chemical screening of the phytochemical group and the colorimetric assays of TPC and TFC were, therefore, carried out in this study. The identification of the phenolic and flavonoid compounds was also attempted in this study by using HPLC. Remarkably, the techniques used in this study to measure the antioxidant activity of plant extracts and groups of compounds produced consistent findings, albeit through their various reaction mechanisms.

In this study, the FRAP and the DPPH assays were tested to pinpoint the antioxidant mechanisms of the examined phytochemicals. The FRAP assay can be employed with human samples and other animal samples [44]. It measures the reduction of the complex of ferric ions (Fe^3+^)-ligand to the ferrous complex (Fe^2+^), based on non-radical single-electron transfer under acidic pH to maintain iron solubility. A single absorption endpoint might not cover the entire reaction since various antioxidants require different durations for the antioxidants and Fe^3+^ to react [44]. It should be stated that the in vitro antioxidant assays have some limitations, such as the fact that they can only measure non-enzymatic antioxidant activity in vitro and may not accurately represent antioxidant activity in vivo or within the human body [51,52]. Due to their weak solubility, low bioavailability, propensity for metabolic breakdown, and quick excretion, phytochemical antioxidants may only be present in trace levels in vivo or systemically. Additionally, metabolites may exhibit increased antioxidant activity compared to their parent molecules [53]. Thus, the pharmacological effects of antioxidation may not only manifest in vivo through their radical scavenging and reducing power shown by DPPH and FRAP assays [51]. The kinetic study of antioxidant capacity may be underestimated. For example, fast-acting antioxidants with few phenol groups were underrated, while slow-acting antioxidants with many phenol groups were given the highest ranks [51]. The targets of the DPPH radical scavenging assays are sterically hindered and stable DPPH radicals. (^•^OH, O_2_^•−^, or lipid oxyl radicals are examples of short-lived radicals that are small and readily accessible in vivo. Therefore, the in vitro assays’ molecular targets and chemistry do not conform to in vivo conditions. The in vitro tests are normally carried out in polar or aqueous phases. It was thus unable to address the lipid oxidation-related radical processes in lipids present in cell membranes [51]. To substantiate the claim of the biological impacts and health advantages of natural antioxidants, further pertinent in vivo activities are required.

Our study found that the plant formula from the root and stem of six mixed herbs showed the highest phenolic content, followed by the extract from *C. digyna*’s root and stem parts. The extracts from *Z. cambodiana*’s root and stem showed the highest TFC, higher than the mixed plant extract. A higher DPPH scavenging activity was, however, found in the root-mixed plant extract and the root of *C. digyna* than in other plant extracts. Considering stem extracts, higher DPPH scavenging activity was found in the *C. digyna* and the stem-mixed plant extract, respectively. The DPPH scavenging effect of the root-mixed plant extract was non-significantly different from gallic acid, a positive control. The highest FRAP value was detected in the mixed plant extract, followed by the *C. digyna* extract when considered in each part. Higher FRAP values were shown in the stem than in the root in the mixed plant and the *C. digyna* extract. Notably, the amount of plant in the formula used for the extraction was approximately five times less than that used in the single plant extraction. However, the antioxidant activity of the mixed plant extract seemed to be amplified. For the first time, our data confirmed the synergistic effect of bioactive compounds in the mixed-plant extract formula that augmented the antioxidative effect.

For phytochemical screening in each plant extract, alkaloids, tannins, flavonoids, and reducing sugar were found in all plants at different levels, but not terpenoids. Terpenoids are a range of compounds taking the role of a metabolic precursor for phytosterol synthesis [54,55]. Some terpenoids reportedly exhibited hormonal effects, including estrogenic effects [56]. However, the bioactivity of terpenoids can vary widely depending on the specific compound and the administered dose. The therapeutic action in women after giving birth after using this medicinal plant formulation following traditional Thai medicine was not caused by the plants’ hormone effect. The most common secondary metabolites proven to have antioxidant properties are polyphenols, such as tannins, saponins, flavonoids, or phenolic compounds [49,57,58]. Their structures are characterized by one or many hydroxyl groups binding to one or more aromatic rings [59]. Tannins have been reported for their antioxidant activity [60,61]. The OH^−^ at the phenyl ring in the tannins structure was involved in antioxidant properties [62] and donated H-atoms to free radicals, generating unreactive or stabilized phenoxyl radicals due to the resonance effect [59]. Flavonoids, a class of organic compounds with diverse phenolic structures, also contributed to antioxidant activity [63]. Flavonoids can scavenge free radical species such as lipid peroxidase (LOO^•^), the hydroxyl radical (^•^OH), and superoxide (O_2_^•−^) by donating the hydrogen atom or an electron [64]. Three partial structures that were involved in the antioxidant activity of flavonoids were (a) the o-dihydroxyl structure in the B ring, (b) the 2,3-double bond with conjugation to a 4-oxo group in ring B, and (c) hydroxyl groups at the 3 and 5 position [65].

The highest DPPH radical scavenging activity and the highest FRAP value of *C. digyna* are consistent with a previous study on the antioxidant activity of the methanol root extract from *C. digyna* [20]. Besides gallic acid, bergenin was previously reported in *C. digyna* to contribute to moderate antioxidant activity in in vitro and in vivo studies [66]. Moreover, isointricatinol, a homoisoflavonoid from the methanol extract of roots, was reported to be attributed to a mild to moderate antioxidant activity based on DPPH and ABTS radical scavenging assays [67]. In our study, both parts of *O. horridus* and *H. cerasoides* exerted the lowest antioxidant activity. On the contrary, previous studies reported that the alcoholic extract of the stem [21] and ethyl acetate extract of stem bark from *H. cerasoides* [68] and the methanolic extract of the stem from *O. horridus* exhibited antioxidant activity [25]. Various and different degrees of activity in the same plant or among medicinal plants might be due to many factors, including the genetic makeup, the time of harvest, the part used, the growth conditions, and the kind of solvent used for the extraction.

Pearson’s correlation analysis and the PCA score plot could unbiasedly scrutinize the most influential compound on antioxidant activity. A strong and significant correlation between TPC and antioxidant activity was corroborated, indicating that TPC in the plant extract was the key determining factor for their antioxidant capacities. Gallic acid (a phenolic compound) and rutin (a flavonoid compound) were the predominant compounds attributed to the antioxidant activity in both Pearson’s correlation analysis and the PCA results. Gallic acid was the predominant compound attributed to the antioxidant activity of the mixed plant extract formula. The most potent antioxidant plant in this study, *C. digyna*, contained the highest gallic acid content compared to other plants. It suggests that gallic acid is a sensitive biomarker in the *C. digyna* extract and the mixed plant extract. This information is pivotal for the quality control of the plant extract and stability evaluation [69].

## 4. Materials and Methods

### 4.1. Materials

Analytical reagent-grade ethanol was purchased from V.S. Chem House (Bangkok, Thailand) for extraction. The phenolic acid standards (≥99% purity), which were gallic acid, protocatechuic acid, vanillic acid, caffeic acid, syringic acid, and ferulic acid, were purchased from Sigma-Aldrich Fine Chemicals (St. Louis, MO, USA). The flavonoid standards (≥99% purity), which were rutin, quercetin, and apigenin, were purchased from Sigma-Aldrich Fine Chemicals (St. Louis, MO, USA). Hydrochloric acid (HCl) 37% was from QReC (Christchurch, New Zealand). Potassium sodium tartrate tetrahydrate (99% purity) was purchased from Merck (Darmstadt, Germany). Methanol (99.9% purity) was purchased from ACL Lab Scan Co. Ltd., (Bangkok, Thailand). 2,2-diphenyl-1-picrylhydrazyl, Iron (III) chloride, and 2,4,6,-tris(2-pyridyl)-S-triazine were purchased from Sigma-Aldrich Co. (St. Louis, MO, USA). Folin–Ciocalteu reagent (Carlo Erba Reagenti, Milan, Italy), sodium carbonate (Na_2_CO_3_), and sodium hydroxide (NaOH) were purchased from Ajax Finechem Pty Ltd. (Auckland, New Zealand) and were used for the total phenolic content analysis.

### 4.2. Plant Sample Preparation

In June 2021, the stems and roots of plants were collected in Roi Et province, Thailand, with a geographic coordinate of 16°3′11″ N 103°39′4″ E. The plant sample was cleaned, damped with clothes, and cut into small pieces before drying in the hot air oven (Contherm Thermotec 2000 Series Ovens, Lower Hutt, New Zealand) at 70 °C for 2 days. After drying, the plant sample was ground into small pieces by roller mill. Each plant (1 kg) was macerated in absolute ethanol (5 L) for 7 days with occasional shaking. The ex-traction was carried out separately for root and stem and repeated twice using the same plant residue. The solvent was pooled, filtered, and then evaporated using a rotary evaporator (IKA, Staufen, Germany). Water was removed to obtain a complete dry extract residue using a freeze dryer (Labconco, Kansas City, MO, USA). In addition, the extract of six mixed plants was also prepared from either root or stem to mimic the formula of traditional Thai medicine. The plant and ethanol ratio was 1 kg of each dry plant powder except for *O. horridus*, which used 0.5 kg (total weight of 5.5 kg), and 27.5 L of ethanol. Accordingly, each plant’s weight in the plant mixture was less than that used for the extraction of a single plant.

### 4.3. Phytochemical Identification

Qualitative phytochemical screening was performed in this study. The major phytoconstituents—tannins, xanthones, terpenoids, steroids, reducing sugar, flavonoids, alkaloids, and saponins—in plant extract were identified, as per the previous reports, with some modifications [70,71,72].

Briefly, for tannin and xanthones, plant extract (0.030 g) was dissolved in 2 mL of ethanol, sonicated at 40 kHz for 5 min with a sonicator (WUC-D22H, Daihan Scientific, Wonju, Republic of Korea), centrifuged at 190× *g* for 10 min, and 1 mL of supernatant was collected. For tannin, 1 mL of 15% ferric chloride (FeCl_3_) was added, and a dark green or blue-black precipitate indicated the presence of tannin. For xanthones, 1 mL of 5% potassium hydroxide was added to the supernatant. A yellow precipitate indicated the presence of xanthones.

For terpenoids, 5 mg of plant extract was dissolved in 1 mL of chloroform, then sonicated at 40 kHz for 5 min and left to dry. Next, 1 mL of 96% sulfuric acid was added and heated to 75 °C in a water bath for 2 min. The gray color indicated the presence of terpenoids.

For steroids, 15 mg of plant extract was dissolved in 1 mL of chloroform and sonicated at 40 kHz for 5 min. Then, the supernatant was added with 1 mL of 96% sulfuric acid. The red-colored lower layer indicated the presence of steroids.

For reducing sugar, a Fehling reagent mixture (Fehling A: Fehling B in a 1:1 ratio) was used. Fehling’s solution A was a mixture containing 0.56 g of copper II sulfate in 8 mL deionized water. Fehling’s solution B comprised 0.8 g sodium hydroxide, 2.8 g potassium sodium tartrate, and 8 mL of deionized water. The 30 mg of plant extract was dis-solved in 2 mL of ethanol, sonicated at 40 kHz for 5 min, and centrifuged at 190× *g* for 10 min. Supernatant in the volume of 1 mL was collected and 1 mL Fehling reagent mixture was added and placed at room temperature for 10 min. A brick-red precipitate indicated the presence of reducing sugar.

For flavonoids, the sodium hydroxide was added into plant extract dissolved in 2 mL of ethanol. The mixture was sonicated at 40 kHz for 5 min, and centrifuged at 190× *g* for 10 min. Then the 1% hydrochloric acid was added into the supernatant of plant extract solution. The clear color change from the yellow color indicated the flavonoid content.

For alkaloids, the 15 mg of plant extract was dissolved in 1 mL of ethanol, sonicated at 40 kHz for 5 min, and centrifuged at 190× *g* for 10 min. The supernatant was then added with 1 mL of 1% hydrochloric acid, followed by 2 drops of Wagner’s reagent (potassium bismuth iodide). A reddish-brown precipitate with turbidity indicated the presence of alkaloids.

Saponins were indicated by the formation of permanent foam at 25 °C after the plant extract was dissolved in 2 mL of deionized water and shaken vigorously by a vortex mixer for 30 s.

A positive outcome indicated that there were phytochemicals present in the extracts based on the levels of color intensity, precipitation, and height of foam formation as com-pared to the control visually (without the crude extract). The outcome was graded on a scale of high (5) to low (1), or absent (0). Therefore, 0 was given when there was no difference from the control. Additionally, the numbers 1, 2, 3, 4, and 5 were given for the slight, small, moderate, more, and maximum change, respectively, in comparison to the control as per a previous study [73].

### 4.4. Identification of Phenolics and Flavonoids in the Extract by HPLC

The components of phenolic and flavonoid contents in plant formula and each plant extract were determined by using HPLC as per the previous study [74]. The phenolic standards used in the experiment were gallic acid, protocatechuic acid, vanillic acid, cinnamic acid, caffeic acid, syringic acid, and ferulic acid. The flavonoid standards were rutin, quercetin, and apigenin. A reverse-phase HPLC system for the analysis was used, comprising Shi-madzu LC-20AC pumps, an SPD-M20A diode array detector, and an InertSustain^®^ C18 column (250 mm × 4.6 mm i.d., 5 µm, GL Sciences Inc., Tokyo, Japan) protected with a guard column. The composition of the solvents and the gradient elution conditions used were described previously [75]. Briefly, the extracts were dissolved in dimethyl sulfoxide (HPLC grade) (final concentration of 1 mg/mL). The sample was filtered through a 0.45 µm membrane filter. A gradient of solvent A (1% acetic acid in water) and solvent B (acetonitrile) was run at a 0.8 mL/min flow rate. Gradient elution was performed as follows: from 0 to 5 min, linear gradient from 5% to 9% solvent B; from 5 to 15 min, 9% solvent B; from 15 to 22 min, linear gradient from 9% to 11% solvent B; from 22 to 38 min, linear gradient from 11% to 18% solvent B; from 38 to 43 min, from 18% to 23% solvent B; from 43 to 44 min, from 23% to 90% solvent B; from 44 to 45 min, linear gradient from 90% to 80% solvent B; from 45 to 55 min, isocratic at 80% solvent B; from 55 to 60 min, linear gradient from 80 to 5% solvent B and a re-equilibration period of 5 min with 5% solvent B used between individual runs. UV-diode array detection was conducted at 280 nm (gallic acid, protocatechuic acid, vanillic acid, and cinnamic acid), 320 nm (caffeic acid, syringic acid, and ferulic acid), and 370 nm (rutin, quercetin, and apigenin). The spectra were recorded from 200 to 600 nm. The samples’ phenolic acid and flavonoid compounds were identified by comparing their relative retention times and UV spectra with those of authentic compounds and detected using an external standard method.

### 4.5. Determination of Total Phenolic Contents

Total phenolic content (TPC) was determined by the Folin–Ciocalteu method according to the method in [76]. Briefly, Folin–Ciocalteu reagent (120 µL) was mixed with 15 µL of each sample (final concentrations ranging from 4–40 µg/mL in methanol) in a 96-well plate. After 5 min, 120 µL of Na_2_CO_3_ solution (60 g/L) was added to each well and mixed. The plate was incubated and protected from light for 90 min before measuring. The absorbance of each well was measured at 725 nm by a microplate reader against a blank (EnSight Multimode plate reader, Waltham, MA, USA). The total phenolic content of the sample was calculated from the standard curve of gallic acid in methanol (y = 0.0485x − 0.0543, R^2^ = 0.9999) across a final concentration range of 2–40 µg/mL). Results are expressed as the gallic acid equivalent (GAE) in mg per gram of dry weight of the extract.

### 4.6. Determination of Total Flavonoid Content

The total flavonoid content (TFC) was determined using the colorimetric method [77]. Briefly, 500 µL of extract samples (stock concentrations of 1 or 2 mg/mL in methanol) was mixed with 2250 µL of distilled water and 150 µL of 5% NaNO_2_ solution. After 6 min, 300 µL of AlCl_3_ (10% *w*/*v*) was added to the solution. Next, the mixture was allowed to stand for 5 min, and 100 µL of NaOH solution (1 M) was added. The absorbance of TFC in a reaction mixture was measured at 510 nm using a spectrophotometer (UV-1700, Shimadzu, Tokyo, Japan). The TFC of the sample was calculated from the standard curve of rutin in methanol (y = 0.0013x + 0.0028, R^2^ = 0.9999) across a final concentration range of 1–500 µg/mL. Results were expressed as µg rutin equivalents (RE) per g (mg RE/g).

### 4.7. DPPH Radical Scavenging Activity

The scavenging activity of the stable 2,2-diphenyl-1-picrylhydrazyl (DPPH) radical was used to measure the antioxidant activity of extracts. First, the solution of DPPH radical in methanol was added to each well of the 96-well plate (a final concentration of 200 μM). Next, various concentrations of extracts dissolved in methanol (0.1–500 μg/mL) were added to a 96-well plate. The microplate was incubated in the dark for 30 min at room temperature and the absorbance was measured at 517 nm using a microplate reader (EnSight Multimode plate reader, Waltham, MA, USA). The concentration of the extract that possessed 50% scavenging activity of DPPH radical was represented as the IC_50_ value and expressed in terms of mean ± SD. Methanol was used as a blank. Gallic acid was used as a positive control in the concentration ranging from 0.1 to 5 μg/mL.

### 4.8. Ferric-Reducing Antioxidant Power (FRAP) Assay

The FRAP reagent was freshly prepared by mixing 300 mM acetate buffer pH 3.6, 10 mM 2,4,6,-tris(2-pyridyl)-s-triazine (TPTZ) solution in 40 mM HCl, 20 mM FeCl_3_ at a ratio of 10:1:1 (*v*/*v*/*v*). The FRAP reagent in a volume of 190 µL was added into each well. Each extract dissolved in methanol at various concentrations (final concentrations ranging from 1 to 25 µg/mL) in the volume of 10 µL was then added to the well. The microplate was incubated in the dark for 30 min at room temperature. The absorbance of Fe^2+^ in each well was measured at 593 nm using a microplate reader (EnSight Multimode plate reader, Waltham, MA, USA). Gallic acid was used as a positive control in the final concentrations ranging from 0.1 to 3 µg/mL. The antioxidant potential of the extracts was determined from ferric-reducing antioxidant power (FRAP). The standard curve was plotted, and the FeSO_4_ in methanol (across a final concentration range of 3.6–90.0 mM) was used to create a linear regression equation (y = 9.4049x + 0.1273, R^2^ = 0.9922) to calculate the FRAP values of the sample represented as the molar concentration of FeSO_4_.

### 4.9. Statistical Analysis

All experiments were performed with at least three replications (n = 3). The results were reported as mean ± standard deviation. The statistical analysis, including regression and Pearson correlation, was performed using IBM SPSS Statistics software (version 27, SPSS Inc., Chicago, IL, USA). One-way ANOVA determined the difference between groups with Tukey’s post hoc test. The *p*-values below 0.05 were considered statistically significant. The principal analysis was built based on the software Visual Studio Code v.1.81.1.

## 5. Conclusions

In this study, the results highlight the antioxidant activity of Thai herbal medicine. The mixed plant extract from the root and stem showed the highest TPC and corresponding high antioxidant activity analyzed by DPPH and FRAP assays. Gallic acid (a phenolic compound) and rutin (a flavonoid compound) were detected in the mixed plant extract. Among single plants, the root and stem of *C. digyna* show the highest antioxidant activity, contributed by the highest TPC and gallic acid. TPC showed a strong positive correlation with antioxidant activity. The principal component analysis revealed that the amounts of gallic acid and rutin are the most influential on antioxidant activity. Moreover, our study provides the information that the mixed plant formula and its components possessed a plausible potential as an antioxidant therapeutic. Our findings support the therapeutic benefits of a single use of the plant in both stem and root form and the synergistic effect when used as a formula. The stem of the medicinal plants in our study can be an alternative to the root, although they yield different antioxidant effects, as using botanical plants’ stems may better aid the sustainable uses of a natural resource than using the root. The examined plants hold promise as sources of natural antioxidant compounds for use in food and medicines to counter oxidative stress such as for puerperium care after parturition. The selected plants and their formula show promise for the upcoming research phases; however, additional relevant chemical structure elucidation of the active phytochemical components and more detailed pharmacological action and safety are required.

## Figures and Tables

**Table 1:** Extraction yield of each part of the plant sample and the extract of the formulation containing six mixed plants.

Scientific Name	Plant’s Abbreviation	%Extraction Yield (*w*/*w*) per Dry Plant	
		Root (R)	Stem (S)
Six mixed plant extract	F	5.46%	3.97%
*Caesalpinia digyna* Rottler	CD	15.82%	6.17%
*Huberantha cerasoides* (Roxb.) Benth	HC	3.17%	3.15%
*Oxyceros horridus* Lour	OH	3.94%	1.56%
*Antidesma ghaesembilla* Gaerth	AG	3.07%	1.77%
*Combretum quadrangulare* Kurz	CQ	3.62%	3.75%
*Ziziphus cambodiana* Pierre	ZC	4.99%	3.40%

**Table 2:** Phenolic and flavonoid contents in the plant extracts based on HPLC analysis.

Compound Content in the Extract (µg/g Dry Extract)	Six Mixed Plants Extract		*Caesalpinia digyna* Rottler		*Huberantha* *cerasoides* (Roxb.) Benth		*Oxyceros* *horridus* Lour		*Antidesma* *ghaesembilla* Gaerth		*Combretum* *quadrangulare* Kurz		*Ziziphus* *cambodiana* Pierre	
	Root	Stem	Root	Stem	Root	Stem	Root	Stem	Root	Stem	Root	Stem	Root	Stem
Gallic acid	401.7 ± 4.3 ^b,A^	617.0 ± 6.0 ^b,B^	735.4 ± 1.3 ^a,A^	1368.1 ± 4.0 ^a,B^	ND	ND	ND	ND	179.1 ± 1.9 ^d,A^	321.8 ± 0.5 ^c,B^	376.2 ± 3.9 ^c,A^	151.9 ± 2.2 ^d,B^	ND	ND
Protocatechuic acid	ND	ND	ND	ND	ND	ND	ND	ND	ND	ND	131.4±4.5	ND	ND	ND
Vanillic acid	ND	98.3 ± 1.8 ^b^	ND	142.8 ± 0.5 ^a^	ND	ND	ND	ND	ND	ND	ND	ND	ND	ND
Caffeic acid	ND	ND	ND	ND	ND	ND	ND	ND	ND	ND	ND	ND	ND	ND
Syringic acid	ND	ND	ND	ND	ND	ND	ND	ND	ND	ND	ND	ND	ND	ND
Ferulic acid	ND	ND	ND	ND	ND	ND	ND	ND	ND	ND	ND	ND	ND	ND
Cinnamic acid	ND	207.2 ± 4.1 ^b^	ND	ND	ND	691.3 ± 4.7 ^a^	109.6 ± 1.6 ^c^	ND	ND	ND	ND	ND	ND	ND
Rutin	92.5 ± 2.6 ^b,A^	139.0 ± 3.6 ^b,A^	ND	ND	111.1 ± 2.9 ^b^	ND	ND	ND	ND	ND	1083.4 ± 17.9 ^a,A^	1373.1 ± 75.6 ^a,B^	ND	ND
Quercetin	ND	96.4 ± 0.8 ^b^	ND	87.1 ± 0.9 ^c^	ND	460.0 ± 17.3 ^a^	ND	ND	ND	ND	ND	ND	ND	ND
Apigenin	ND	151.1 ± 0.8 ^b^	97.5 ± 2.9 ^a^	ND	ND	1344.3 ± 3.0 ^a^	ND	ND	ND	ND	ND	ND	ND	ND

ND = Not detected. Results are presented as the mean ± SD (n = 3). Different lowercase letters indicate a significant difference in compound content among plants in the same row at *p* < 0.05. Different uppercase letters indicate a significant difference in compound content among parts of the same plant in the same row at *p* < 0.05. Means with different letters differ significantly (*p* < 0.05). Per gram extract in the stem of *A. ghaesembilla* (328.8 ± 16 µg of GAE) was not significantly different from the stem extract of *Z. cambodiana* (313.8 ± 7.4 µg of GAE) (*p* > 0.05).

**Table 3:** Total phenolic and flavonoid contents and antioxidant activity in the plant extracts.

Plant Extract	TPC		TFC		DPPH		FRAP	
	(mg of Gallic Acid/g of Dry Extract)		(mg of Rutin/g of Dry Extract)		(IC_50_ of DPPH (µg/mL))		(FeSO_4_ Equivalent (mM/g of Dry Extract))	
	Root	Stem	Root	Stem	Root	Stem	Root	Stem
Six mixed plants extract	1682.7 ± 19.8 ^a,A^	1006.3 ± 8.6 ^a,B^	23.0 ± 0.1 ^d,A^	23.0 ± 0.4 ^c,A^	3.8 ± 0.2 ^a,A^	10.0 ± 0.3 ^a,A^	32.3 ± 1.3 ^a,A^	19.0 ± 1.0 ^b,B^
*Caesalpinia digyna Rottler*	1085.8 ± 20.5 ^b,A^	661.4 ± 33.0 ^b,B^	24.7 ± 0.2 ^c,A^	22.8 ± 0.3 ^c,B^	7.8 ± 0.3 ^a,A^	5.8 ± 0.1 ^a,A^	10.7 ± 0.5 ^b,A^	9.3 ± 0.5 ^c,B^
*Huberantha cerasoides* (Roxb.) Benth	138.4 ± 0.9 ^f,A^	120.2 ± 2.1 ^e,A^	11.5 ± 0.1 ^f,A^	12.8 ± 0.1 ^e,B^	172.9 ± 4.4 ^d,A^	267.3 ± 5.7 ^d,B^	1.8 ± 0.1 ^f,A^	2.4 ± 0.0 ^f,g,A^
*Oxyceros horridus* Lour	95.1 ± 2.9 ^g,A^	55.5 ± 1.0 ^f,B^	9.7 ± 0.1 ^g,A^	4.7 ± 0.2 ^f,B^	257.1 ± 8.2 ^e,A^	inactive	2.2 ± 0.1 ^e,f,A^	1.3 ± 0.1 ^g,A^
*Antidesma ghaesembilla* Gaerth	396.4 ± 6.4 ^c,A^	328.8 ± 16.0 ^c,B^	30.0 ± 0.1 ^b,A^	27.1 ± 0.7 ^b,B^	32.3 ± 2.4 ^b,A^	45.6 ± 0.6 ^b,B^	6.8 ± 0.4 ^c,A^	7.0 ± 0.2 ^d,A^
*Combretum quadrangulare* Kurz	190.5 ± 4.5 ^e,A^	222.3 ± 6.9 ^d,B^	15.4 ± 0.2 ^e,A^	10.4 ± 0.3 ^d,B^	74.8 ± 1.1 ^c,A^	57.8 ± 2.0 ^c,B^	3.1 ± 0.2 ^e,A^	3.0 ± 0.1 ^f,A^
*Ziziphus cambodiana* Pierre	259.7 ± 8.2 ^d,A^	313.8 ± 7.4 ^c,B^	39.3 ± 0.8 ^a,A^	54.9 ± 1.0 ^a,B^	38.9 ± 0.7 ^b,A^	47.8 ± 1.4 ^b,B^	5.0 ± 0.1 ^d,A^	5.6 ± 0.1 ^e,A^
Gallic acid					3.4 ± 0.1 ^a^		31.5 ± 1.8 ^a^	

Inactive means when a half-maximal inhibition of DPPH radical was not obtained even when using a maximum concentration. TPC = total phenolic content; TFC = total flavonoid content; DPPH = 2,2-diphenyl-1-picrylhydrazylradical scavenging activity; FRAP = ferric reducing antioxidant power. Results are presented as the mean ± SD (n = 3). Different lowercase letters indicate a significant difference in compound content among plants between rows at *p* < 0.05. Different uppercase letters indicate a significant difference in compound content among parts of the same plant in the same row at *p* < 0.05. Means with different letters differ significantly (*p* < 0.05).

## Data Availability

Not applicable.

## Supplementary Materials

The following supporting information can be downloaded at: https://www.mdpi.com/article/10.3390/ijms241713425/s1.
