# Supplementary material for: Phytochemical Screening on Phenolic, Flavonoid Contents, and Antioxidant Activities of Six Indigenous Plants Used in Traditional Thai Medicine
Source: Int J Mol Sci. 2023 Aug 30;24(17):13425. doi: 10.3390/ijms241713425 (PMC10487580; doi:10.3390/ijms241713425)
Supplement: Supplementary file 1 [file ijms-24-13425-s001.zip › ijms-2555934-supplementary.pdf]

Supplementary Material

Article

Phytochemical Screening on Phenolic, Flavonoid Contents, and Antioxidant Activities of Six Indigenous Plants Used in Traditional Thai Medicine

Tanawuth Tiranakwit [1], Wimonwan Puangpun [2], Kawintra Tamprasit [3], Natthapong Wichai [4], Sirithon Siriamornpun [5], Tarapong Srisongkram [3,6] and Natthida Weerapreeyakul [3,6,*]

[1] Program of Pharmaceutical Sciences, Graduate School, Faculty of Pharmaceutical Sciences, Khon Kaen University, Khon Kaen 40002, Thailand
[2] Program of Doctor of Pharmacy, Faculty of Pharmaceutical Sciences, Khon Kaen University, Khon Kaen 40002, Thailand
[3] Human High Performance and Health Promotion Research Institute, Khon Kaen University, Khon Kaen 40002, Thailand
[4] Department of Pharmaceutical Sciences, Faculty of Pharmacy, Mahasarakham University, Maha Sarakham 44150, Thailand
[5] Research Unit of Thai Food Innovation, Department of Food Technology and Nutrition, Faculty of Technology, Mahasarakham University, Maha Sarakham 44150, Thailand
[6] Division of Pharmaceutical Chemistry, Faculty of Pharmaceutical Sciences, Khon Kaen University, Khon Kaen 40002, Thailand
* Correspondence: natthida@kku.ac.th

Supplementary Table S1. Phytochemicals screening test for tannins, xanthones, terpenoids, steroids, reducing sugar, flavonoids, alkaloids, and saponin.

Phytochemicals	Positive results	
	Description	Color/Precipitate/Foam
Tannin	Dark green or blue-black precipitate	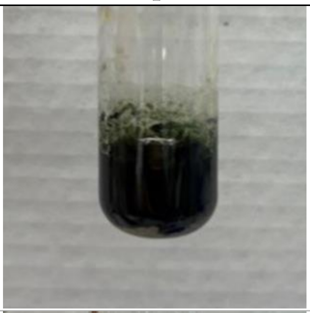
Xanthones	Yellow precipitate	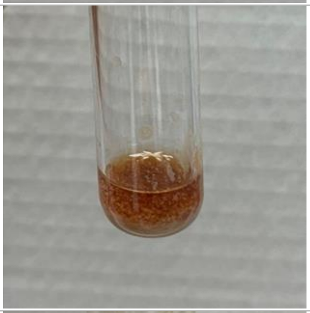
Terpenoids	Gray color	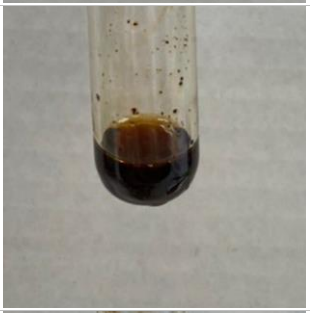
Steroids	Red color in the lower layer	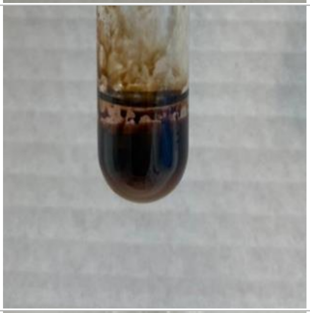
Reducing sugar	Brick-red precipitate.	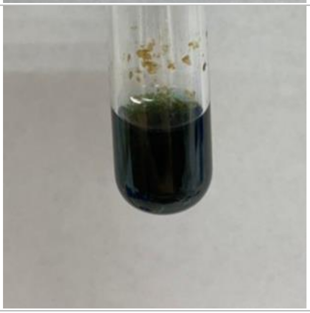

Phytochemicals	Positive results	
	Description	Color/Precipitate/Foam
Flavonoids	Yellow color	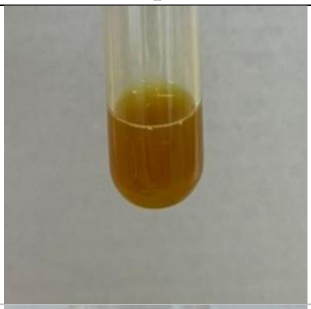
Alkaloids	Reddish-brown precipitate with turbidity	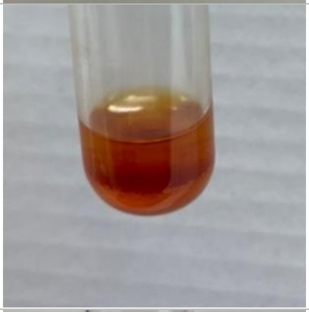
Saponins	Formation of permanent foam	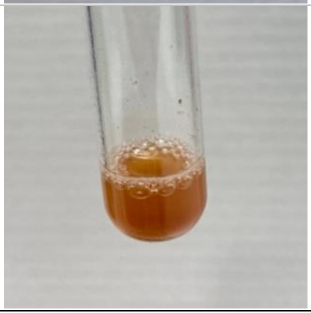

Supplementary Table S2. Detection wavelength and retention times of standard phenolic and flavonoid compounds (n = 3).

Standard compounds	Retention time (min)	Wavelength (nm)
**Hydroxybenzoic acids**		
Gallic acid (1)	7.549	280
Protocatechuic acid (2)	13.382	280
Vanillic acid (3)	25.390	280
Syringic acid (5)	30.340	320
**Hydroxycinnamic acids**		
Caffeic acid (4)	27.341	320
Ferulic acid (6)	45.201	320
Cinnamic acid (7)	53.225	280
**Flavonoid compounds**		
Rutin (8)	48.363	370
Quercetin (9)	52.985	370
Apigenin (10)	53.506	370

(A)

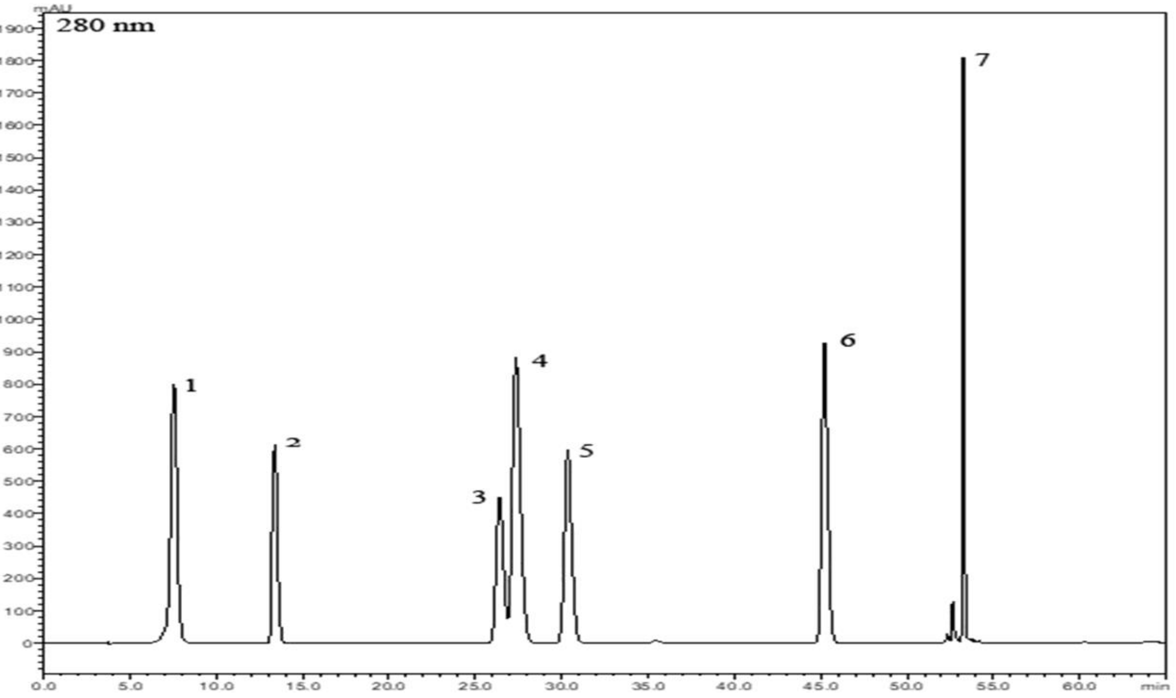

(B)

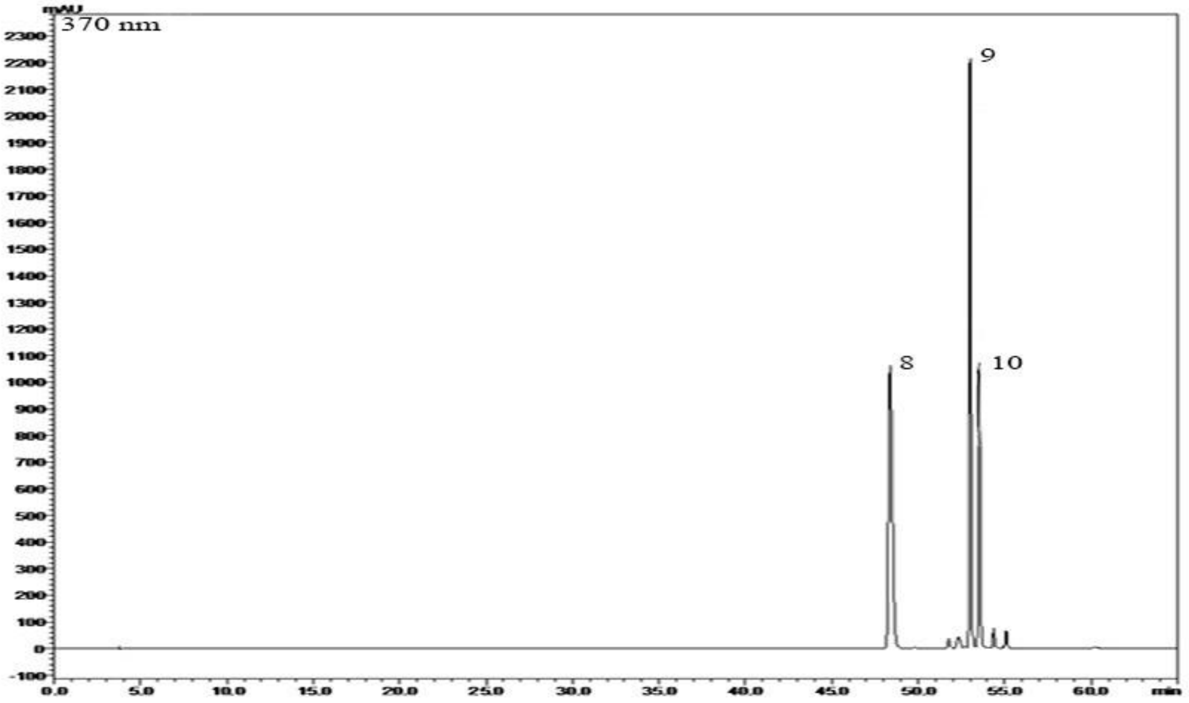

Figure S1. Chromatograms showing the peaks of phenolic and flavonoid compounds obtained for the mixture containing all phenolic compounds (A) and flavonoid compound (B) at 280 nm and 370 nm. The peaks represent the following phenolics: 1=gallic acid, 2=protocatechuic acid, 3=vanillic acid, 4=caffeic acid, 5=syringic acid, 6=ferulic acid, 7=cinnamic acid, 8=rutin, 9=quercetin, 10=apigenin.

(A) Root **(B) Stem**

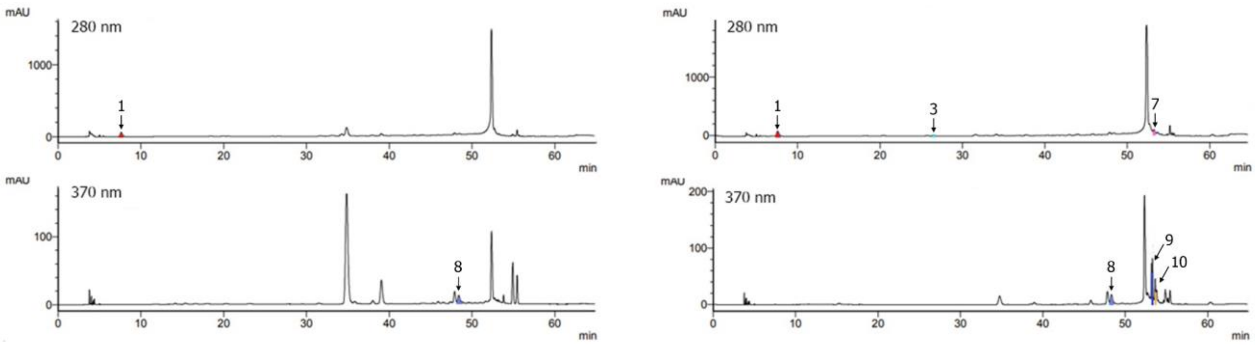

Figure S2. HPLC chromatogram fingerprint of ethanolic extract from (A) the root and (B) the stem of the mixed plant extract showing mixed plant extract at two different wavelengths (280 nm and 370 nm, respectively). (A) Chromatograms show the peaks of gallic acid (1) at 7.526 min (280 nm), rutin (8) at 48.397 min (370 nm), and the unknown peaks. (B) Chromatograms show the peaks of gallic acid (1) at 7.579 min (280 nm), vanillic acid (3) at 26.479 min (280 nm), cinnamic acid (7) at 53.204 min (280 nm), rutin (8) at 48.315 min (370 nm), quercetin (9) at 53.293 min (370 nm), apigenin (10) at 53.636 min (370 nm), and the unknown peaks.

(A) Root **(B) Stem**

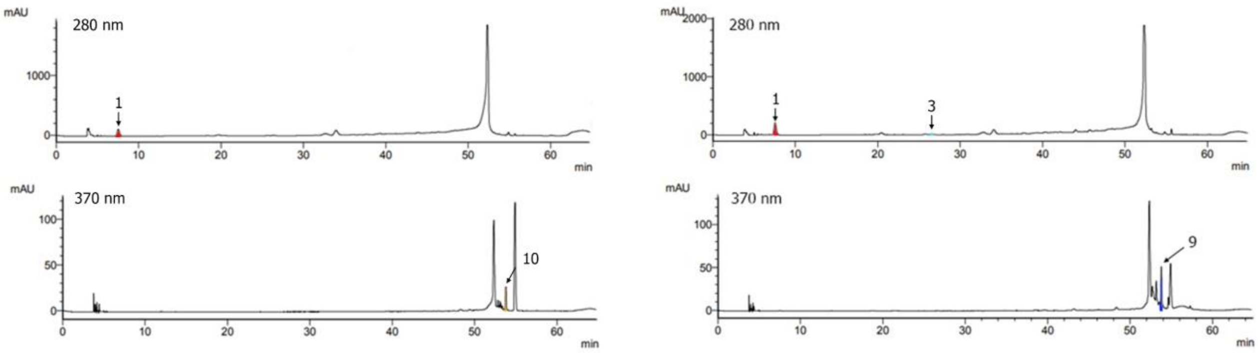

Figure S3. HPLC chromatogram fingerprint of ethanolic extract from (A) the root and (B) the stem of *C. digyna* showing at two different wavelengths (280 nm and 370 nm, respectively). (A) Chromatograms show the peaks of gallic acid (1) at 7.526 min (280 nm), apigenin (10) at 53.774 min (370 nm), and the unknown peaks. (B) Chromatograms show the peaks of gallic acid (1) at 7.547 min (280 nm), vanillic acid (3) at 26.519 min (280 nm), quercetin (9) at 53.16 min (370 nm), and the unknown peaks.

(**A**) **Root** (**B**) **Stem**

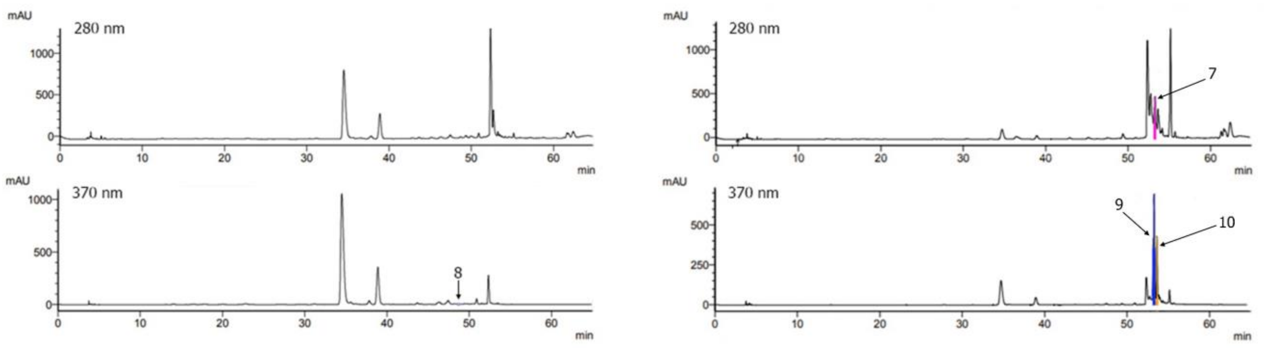

Figure S4. HPLC chromatogram fingerprint of ethanolic extract from (A) the root and (B) the stem of *H. cerasoides* showing at two different wavelengths (280 nm and 370 nm, respectively). (A) Chromatograms show the peaks of rutin (8) at 48.684 min (370 nm), and the unknown peaks. (B) Chromatograms show the peaks of cinnamic acid (7) at 53.265 min (280 nm), quercetin (9) at 53.267 min (370 nm), apigenin (10) at 53.661 min (370 nm), and the unknown peaks.

(**A**) **Root** (**B**) **Stem**

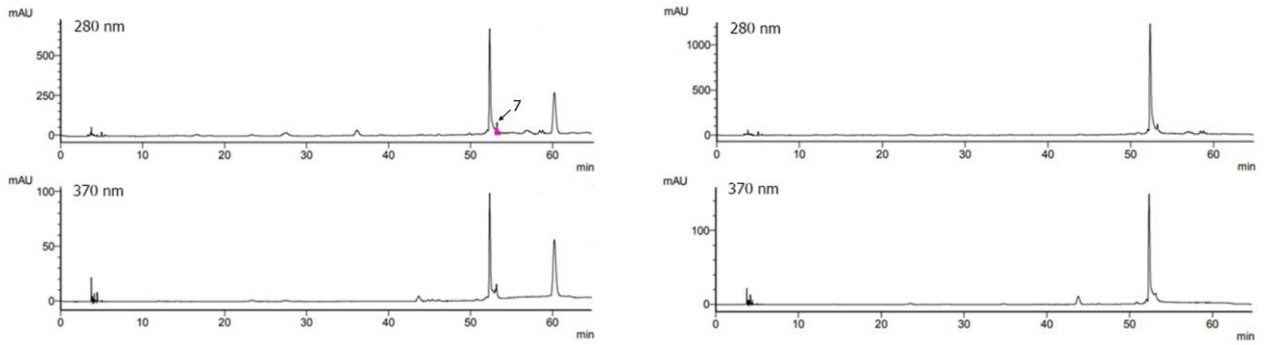

Figure S5. HPLC chromatogram fingerprint of ethanolic extract from (A) the root and (B) the stem of *O. horridus* showing at two different wavelengths (280 nm and 370 nm, respectively). (A) Chromatograms show the peaks of cinnamic acid (7) at 53.221 (280 nm) and the unknown peaks. (B) Chromatograms show the peaks of the unknown peaks.

(A) Root **(B) Stem**

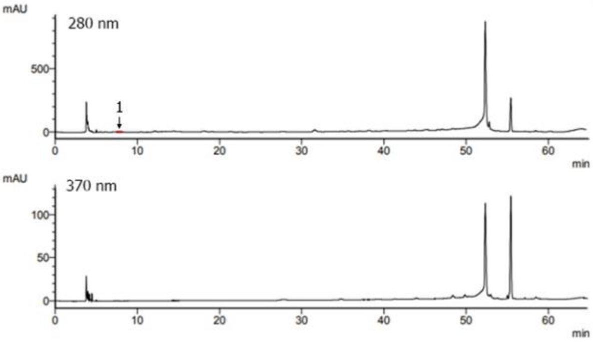

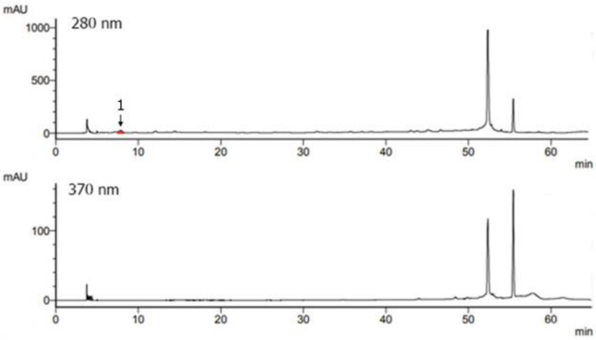

Figure S6. HPLC chromatogram fingerprint of ethanolic extract from (A) the root and (B) the stem of *A. ghaesembilla* showing at two different wavelengths (280 nm and 370 nm, respectively). (A) Chromatograms show peaks of gallic acid (1) at 7.887 min (280 nm) and the unknown. (B) Chromatograms show the peaks of gallic acid (1) at 7.879 min (280 nm) and the unknown peaks.

(A) Root **(B) Stem**

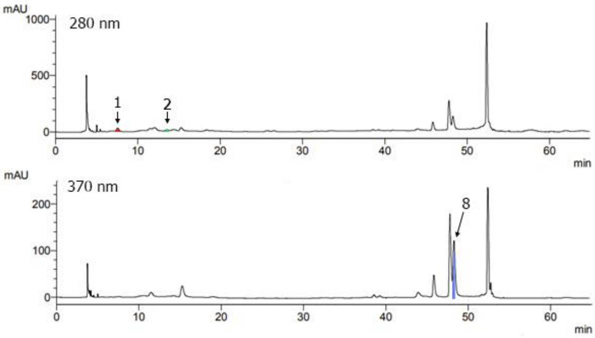

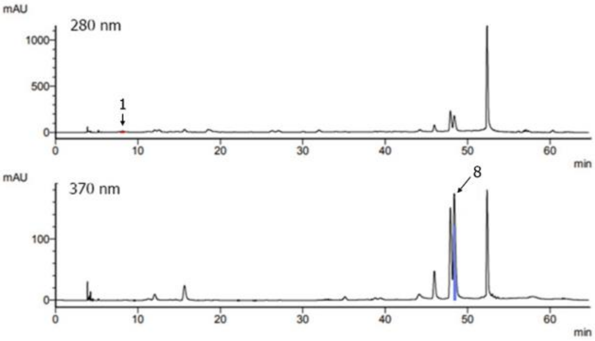

Figure S7. HPLC chromatogram fingerprint of ethanolic extract from (A) the root and (B) the stem of *C. quadrangulare* showing at two different wavelengths (280 nm and 370 nm, respectively). (A) Chromatograms show the peaks of gallic acid (1) at 7.542 min (270 nm), protocatechuic acid (2) at 13.557 min (280 nm), rutin (8) at 48.258 min (370 nm), and the unknown peaks. (B) Chromatograms show the peaks of gallic acid (1) at 7.856 min (270 nm), rutin (8) at 48.377 min (at 380 nm), and the unknown peaks.

(A) Root **(B) Stem**

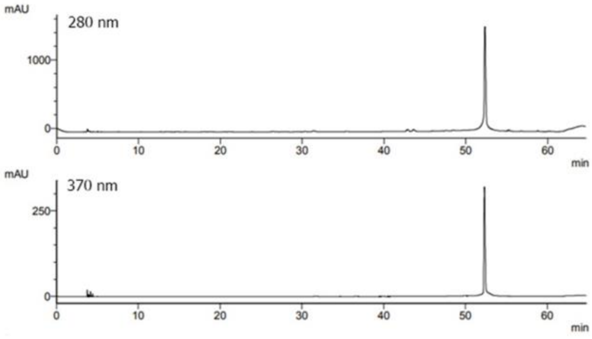

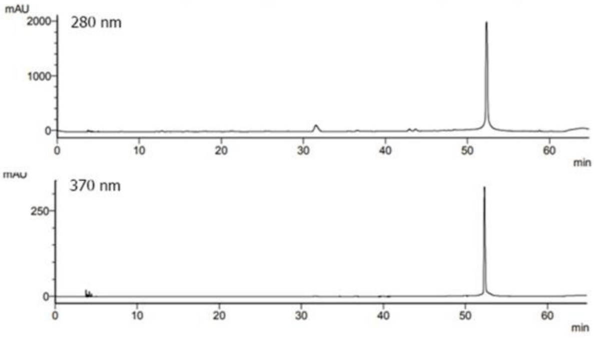

Figure S8. HPLC chromatogram fingerprint of ethanolic extract from (A) the root and (B) the stem of *Z. cambodiana* showing the unknown peaks at two different wavelengths (280 nm and 370 nm, respectively).
